# An Investigation of Social Status among Adolescents and Young Adults Who Have Been Diagnosed with Cancer in Canada

**DOI:** 10.3390/cancers15133436

**Published:** 2023-06-30

**Authors:** Fiona S. M. Schulte, Sharon H. J. Hou, Jacqueline L. Bender, Joshua Tulk, Amanda Wurz, Anika Petrella, Catherine M. Sabiston, Norma D’Agostino, Karine Chalifour, Geoff Eaton, Sheila N. Garland

**Affiliations:** 1 Department of Oncology, Division of Psychosocial Oncology, Cumming School of Medicine, University of Calgary, Calgary, AB T2N 1N4, Canada; sharon.hou@ucalgary.ca; 2 Department of Supportive Care, Princess Margaret Cancer Centre, Toronto, ON M5G 2M9, Canada; jackie.bender@uhnresearch.ca (J.L.B.); norma.d'agostino@uhn.ca (N.D.); 3 Dalla Lana School of Public Health, University of Toronto, Toronto, ON M5G 2C1, Canada; 4 Department of Psychology, Faculty of Science, Memorial University, St. John’s, NL A1B 3X9, Canada; jgjtulk@mun.ca (J.T.); sheila.garland@mun.ca (S.N.G.); 5 Discipline of Oncology, Faculty of Medicine, Memorial University, St. John’s, NL A1B 3V6, Canada; 6 School of Kinesiology, University of the Fraser Valley, Chilliwack, BC V2R 0N3, Canada; amanda.wurz@ufv.ca; 7 Cancer Clinical Trials Unit, University College Hospital, London WC1V 6LJ, UK; anika.r.petrella@gmail.com; 8 Department of Exercise Sciences, Faculty of Kinesiology & Physical Education, University of Toronto, Toronto, ON M5S 2C9, Canada; catherine.sabiston@utoronto.ca; 9 Young Adult Cancer Canada, St. John’s, NL A1B 3K3, Canada; karine@youngadultcancer.ca (K.C.); geoff@youngadultcancer.ca (G.E.)

**Keywords:** adolescent and young adult, neoplasms, social status, survivorship

## Abstract

**Simple Summary:**

Adolescents and young adults (AYAs) diagnosed with cancer are a particularly vulnerable patient population. Cancer and the long-term effects of treatment can impact a young person’s ability to progress through typical developmental stages, negatively impacting their social status including education, employment, relationships status and independent living. The study aims were to: (1) compare social status among AYAs diagnosed with cancer to a community population; (2) describe AYAs’ change in employment/education status; and (3) examine predictors of social status. We showed that AYAs diagnosed with cancer were less likely to be employed and more likely to be living at home with parents when compared to a community sample. These challenges may have many long-term financial and quality of life implications for these patients.

**Abstract:**

Background: Aims were to: (1) compare social status among AYAs diagnosed with cancer to a community population; (2) describe AYAs’ change in employment/education status; and (3) examine predictors of social status. Method: Social status (i.e., education, employment, relationship status, and living arrangement) was captured from young adults diagnosed with cancer recruited via social media through a community-based organization from across Canada and randomly matched to a community sample by sex, age, province of residence, total household income and race/ethnicity at a ratio of 1:3. Results: AYAs with cancer (N = 622) were an average of 4.45 (SD = 5.42) years from the completion of treatment and were less likely to be employed (χ^2^ = 96.35, *p* < 0.001) and more likely to be living at home with parents (χ^2^ = 17.00, *p* < 0.001). There were no differences in education or relationship status. Overall, 41% and 45% of AYAs reported quitting school or work, respectively. Non-metastatic disease (AOR 3.23, 95% CI 1.08–9.62), and better physical (AOR 1.07 95% CI 1.04–1.10) and mental quality of life (QOL)(AOR 1.06 95% CI 1.03–1.09), were associated with employment. Worse mental QOL (AOR 1.04 95% CI 1.01–1.07), less post-traumatic growth (AOR 1.01 95% CI 1.00–1.03), and social support (AOR 0.27, 95% CI 0.18–0.41) were associated with being single. Non-White race (AOR 3.19 95% CI 1.02–9.97) and less post-traumatic growth (AOR 0.97 95% CI 0.95–0.99) were associated with living with parents. Conclusions: AYAs diagnosed with cancer experience differences in attainment of employment and independent living compared to a community sample. These challenges may have implications for physical and mental QOL.

## 1. Introduction

Approximately 8000 adolescent and young adults (AYAs) (15–39 years of age) are diagnosed with cancer each year in Canada [1,2]. Survival rates in this population are relatively high, and as a result, the majority of AYAs diagnosed with cancer will live 50–60 years beyond their diagnosis and treatment [3]. However, AYAs are a particularly vulnerable patient population given the critical time at which they are diagnosed, which has been shown to have negative impacts on quality of life (QOL) [4]. Specifically, AYAs are diagnosed during a time in their development characterized by the initiation of identity formation, the emergence of independence, educational attainment, and exploration of careers and relationships. Cancer and the long-term effects of treatment can impact a young person’s ability to progress through typical developmental stages, negatively impacting their social status. Social status is a critical indicator of one’s functioning within society and is an important metric to understand the extent to which AYAs diagnosed with cancer are achieving specific milestones at the same rate as their peers. Examples of important indicators include educational achievement, employment, relationship status, and living status. Ultimately, the delay or inability to participate in key milestones could have severely negative consequences for AYAs. 

There has been a paucity of research focused on the social status of AYAs diagnosed with cancer. Instead, the majority of research focused on social status has centered on survivors of childhood cancer. This literature is relatively robust with strong evidence indicating that survivors of childhood cancer are at risk of disruptions to social status, including educational attainment, employment, and relationship status [5,6]. For the most part, this literature has linked poor social status to cognitive deficits resulting from toxic therapies directed at the central nervous system during key developmental periods. Recent international guidelines proposed for child, adolescent, and young adult survivors suggest that education and employment status is impaired for these youth compared to controls and call for the need for regular surveillance of education and employment status [5]. These guidelines were limited, however, in that they only considered youth diagnosed <30 years of age, which does not account for the full scope of AYAs diagnosed with cancer. 

Research that has been conducted with AYAs typically reports negative social status. For example, a large population-based case–control registry study in the Netherlands concluded that AYAs 18–39 years of age at diagnosis were significantly less often employed compared with controls [7]. Registry data from the U.S. have shown that AYAs 18–37 years of age at diagnosis were less likely to be married and, for those who were married, at an increased risk of being separated or divorced than controls [8]. These studies have been limited, however, by a lack of a consistent definition of AYAs. In particular, the exclusion of patients diagnosed as adolescents (15–17 years of age) from these data is misleading, as these youth are known to have inferior outcomes compared to other age cohorts [9,10,11]. In addition, data examining social status are lacking from a Canadian sample. 

Thus, the aims of the current study were to: (1) compare social status (i.e., education/employment status, relationship status, living arrangement) among a sample of AYAs diagnosed with cancer between 15–39 years old recruited from across Canada to a community population sample matched by sex, age, province of residence, total household income, and race/ethnicity; (2) to describe perceived change to social status as a result of cancer and its treatment among AYAs diagnosed with cancer recruited from across Canada; and (3) examine demographic (e.g., sex, race/ethnicity, age), clinical (e.g., diagnosis, treatment), and psychosocial predictors (e.g., physical and mental quality of life, post-traumatic growth, and social support) of social status. We hypothesized that AYAs diagnosed with cancer would report significantly different social status compared to their peers without cancer. 

## 2. Materials and Methods

The data for the current study are from the Young Adults with Cancer in their Prime (YACPRIME) Study, which was a cross-sectional survey. The YACPRIME study is a collaborative patient-oriented research project conducted in partnership with Young Adult Cancer Canada (YACC), the leading support and advocacy organization devoted to young adults living with, through, and beyond cancer. The aim of the YACPRIME study was to provide a comprehensive picture of the needs of YAs with cancer in Canada and was co-developed by YACC and the research team. YACC was central to the development of the study and study design, as well as the distribution of the survey to AYAs across Canada. Monthly meetings were held with all members of the research team (patients, public, researchers) to: revisit key priorities, provide feedback on key variables of interest for investigation, provide interpretation of key findings, provide direction for additional analyses, and strategize regarding knowledge dissemination.

### 2.1. Participants 

To be eligible to participate in the YACPRIME Study, AYAs needed to have a self-reported diagnosis of cancer between the ages of 15 and 39 years, consistent with the Canadian Framework for the Care and Support of Adolescents and Young Adults [3], currently be 19 years of age or older to consent to participate, and reside in Canada. The survey was available in both English and French.

AYAs diagnosed with cancer were assessed relative to a comparison group derived from the Canadian Community Health Survey (CCHS), a national cross-sectional survey of the Canadian population that collects information related to health status, health care utilization, and health determinants from Canadians aged 12 and over who live in private dwellings in all ten provinces and three territories. The CCHS is a joint effort of Health Canada, the Public Health Agency of Canada, Statistics Canada, and the Canadian Institute for Health Information (CIHI). An area frame and multistage stratified cluster-sampling procedure was used to survey 63,522 households across Canada [12]. Excluded from the target population were persons living on reserves, full-time members of the Canadian Forces, institutionalized populations, children in foster care, and certain remote regions. Such exclusions account for less than 3% of the total Canadian population. CCHS data are available for public use. Data from the 2017–2018 CCHS cycle were used for the current study. Given that this was a previously collected cohort, we will refer to it as a comparison group rather than a control group. For the purposes of the current study, we excluded people with a past or current history of cancer from the comparison group.

### 2.2. Recruitment

Participants were recruited online via social media through YACC. No compensation was provided as part of survey completion. 

### 2.3. Procedure

Following informed consent, participants completed the YACPRIME survey online. Patient partners from YACC played a crucial role in helping to recruit participants through direct emails, media promotion, online advertisements, and social media posts. The study officially opened in June 2017 and closed March 2018.

### 2.4. Measures

#### 2.4.1. Social Status

Social status was asked in the YACPRIME study using the following questions: What is your current primary school/employment status? How would you describe your relationship status? How would you describe your living situation? To address our second aim, the survey participants were asked: “What was the biggest change to your school/employment status because of your cancer or its treatment” with the following response items: “it has not changed because of my cancer or it’s treatment”; “I quit working completely”; “I quit going to school completely”; “I changed my work status from full to part-time”; “I changed my school status from full to part-time”; “I took more than 2 weeks total time off from work”; “I took more than 2 weeks total time off from school”. 

#### 2.4.2. Psychosocial Well-Being

Psychosocial well-being was assessed using measures of Quality of Life (SF-12, physical [PCS] and mental health component scores [MCS]) [13], Post-traumatic Growth (Post-Traumatic Growth Inventory [PTGI]) [14], and Social Support (Medical Outcomes Study Social Support Survey [MOS-SSS]) [15]. For the PTGI, participants were asked to indicate for each of the statements the degree to which changes occurred as a result of their cancer experience. Higher scores on all these measures reflect more positive outcomes. 

#### 2.4.3. Demographic and Clinical Data

Demographic (i.e., sex [male/female], race/ethnicity [White/other], age, province of residence and total household income), clinical (diagnosis [blood cancer/other], and clinical data (diagnosis [blood cancer/other], treatment [chemotherapy [yes/no], metastatic [yes/no]),) were collected as part of the YACPRIME survey.

### 2.5. Statistical Analysis

Given some of the unique characteristics of our sample (i.e., large proportion of females, younger sample), our comparison sample was randomly matched to the YACPRIME sample by sex [male, female], age [20 and 24 years; 25 to 29 years; 30 to 34 years; 35 to 39 years; 40 to 44 years; 45–49 years; 50–54 years; 55–59 years; 60–64 years] province of residence, total household income [less than CAD 20,000; CAD 20,000 to less than CAD 40,000; CAD 40,000 to less than CAD 60,000; CAD 60,000 to less than CAD 80,000; >CAD 80,000)], and race/ethnicity [white, non-white] at a ratio of 1:3. After matching to a case, the controls were censored so that they could not be assigned to another case. A total of 1688 comparisons were identified. To facilitate matching across social status, outcomes were collapsed to reflect the following categories: employed (yes/no); student (yes/no); single (yes/no); and living at home (yes/no).

Demographic characteristics were summarized and compared between AYAs diagnosed with cancer and comparisons using t-tests or chi-square analyses where appropriate. To address our first aim, chi-square analyses were conducted to explore differences in social status between comparisons and AYAs diagnosed with cancer achieving social status milestones. Acknowledging the many changes that can take place over this age range, the sample was also stratified by age to examine differences in social status across age trajectories. To address our second aim, descriptive statistics (frequencies) were used to describe change in employment/education status for AYAs as a result of their cancer diagnosis or treatment. Finally, to address our third aim, multivariable logistic regression analyses were conducted to examine predictors of social status. Student status was eliminated from these analyses due the confounding influence of age. Demographic and clinical data covering race/ethnicity (White/other), age (continuous variable), diagnosis (blood/other), treatment (chemotherapy yes/no), and metastatic status (yes/no) were included along with psychosocial variables (i.e., post-traumatic growth, social support, physical and mental quality of life, continuous variables). The selection of predictors was based on the literature and our own assumptions. Adjusted odds ratios (AOR) and 95% confidence intervals (CI) were reported. All analyses were conducted in SPSS version 26. AYAs diagnosed with cancer were consulted during the analysis to provide feedback and interpretations of key findings. 

## 3. Results

### 3.1. Characteristics of the Sample

Descriptive characteristics of our sample (*n* = 622) can be found in Table 1. Eighty-six percent of the sample identified as female. The majority (60.6%) of AYA cancer patients were 30–39 years at the time of study and an average of 4.45 (SD = 5.42) years from the completion of treatment. The most common diagnoses were blood cancers (27.8%), followed by breast cancer (27.3%). The survey was completed in French by 6.3% (*n* = 39).

### 3.2. Aim 1: Social Status in AYAs Diagnosed with Cancer and a Comparison Sample

**Figure 1:**
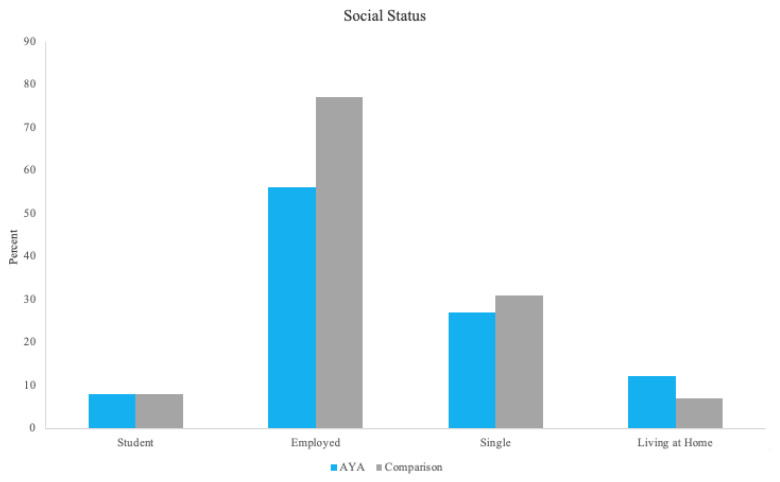
Social status in AYAs diagnosed with cancer and a comparison sample.

**Figure 2:**
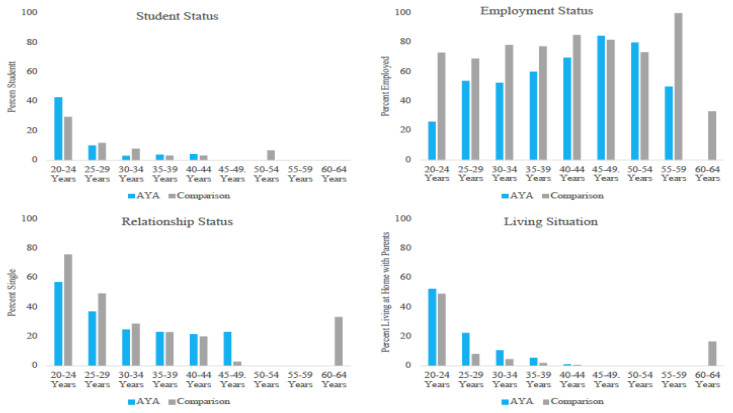
Social status in AYAs diagnosed with cancer and a comparison sample by age trajectories.

There were significant differences between AYAs diagnosed with cancer and comparisons on social status outcomes of *employment status* (χ^2^ = 96.35, *p* < 0.001) and *living situation* (χ^2^ = 17.0, *p* < 0.001), with AYAs diagnosed with cancer being significantly more likely to be unemployed and living at home than comparisons (Figure 1). Findings from the stratified analyses are described below and depicted in Figure 2. 

#### 3.2.1. Education Status

There were no differences in education status across the age trajectories, with the exception of 30–34 years, where comparisons were significantly more likely to be students than AYAs (χ^2^ = 4.42, *p* < 0.05).

#### 3.2.2. Employment Status

AYAs diagnosed with cancer were unemployed at a significantly higher rate than comparisons until 45 years of age (20–24 years: χ^2^ = 27.78, *p* < 0.001; 25–29 years: χ^2^ = 6.61, *p* < 0.01; 30–34 years: χ^2^ = 45.94, *p* < 0.001; 35–39 years: χ^2^ = 19.69, *p* < 0.001; 40–44 years: χ^2^ = 10.56, *p* < 0.001), at which point significant differences were no longer noted. 

#### 3.2.3. Relationship Status

There was a significant difference between AYAs diagnosed with cancer and comparisons in terms of relationship status from 20–29 years of age (20–24 years: χ^2^ = 5.15, *p* < 0.05; 25–29 years: χ^2^ = 4.04, *p* < 0.05), with AYAs less likely to be single than comparisons. At age 45–49 years, AYAs were significantly more likely to be single (χ^2^ = 4.72, *p* < 0.05). 

#### 3.2.4. Living Situation

From 25–39 years of age, AYAs diagnosed with cancer lived at home with parents at a significantly higher rate than comparisons (25–29 years: χ^2^ = 12.74, *p* < 0.001; 30–34 years: χ^2^ = 8.99, *p* < 0.01; 35–39 years: χ^2^ = 4.89, *p* < 0.05). 

### 3.3. Aim 2: Change in Education and Employment Status

Of the total YACPRIME sample, 15.8% (*n* = 98) reported going to school either full-time or part-time and 66.1% (*n* = 411) reported working either full- or part-time prior to their cancer diagnosis. The remainder were unemployed (3.4%), full-time homemakers or family caregivers (5.6%), or ‘other’ (9.2%). Of those in school prior to their cancer diagnosis, 41% (*n* = 40) reported quitting school completely, 12% (*n* = 12) reported changing their status from full-time to part-time, and 43% (*n* = 42) reported taking >2 weeks off school as a result of their cancer and/or treatment. Of those working prior to their cancer diagnosis, 45% (*n* = 183) reported quitting work completely, being let go, or going on long-term disability; 8% (*n* = 32) reported changing their work status from full- to part-time; and 53% (*n* = 218) reported taking >2 weeks off work as a result of their cancer and/or treatment. In total, only 10% of the sample (*n* = 62) reported that their education or employment status had not changed as a result of cancer or its treatment. 

### 3.4. Aim 3: Predictors of Social Status

Results of the multivariable logistic regression can be found in Table 2. 

#### 3.4.1. Employment Status

Older age (AOR 1.07 95% CI 1.02–1.12) and non-metastatic cancer stage (AOR 3.23, 95% CI 1.08–9.62), as well as higher physical (AOR 1.07 95% CI 1.04–1.10) and mental quality of life (AOR 1.06 95% CI 1.03–1.09), were significantly associated with being employed.

#### 3.4.2. Relationship Status

Factors associated with being single included younger age (AOR 0.89, 95% CI 0.84–0.94), poorer mental quality of life (AOR 1.04 95% CI 1.01–1.07), less post-traumatic growth (AOR 1.01 95% CI 1.00–1.03), and lower levels of social support (AOR 0.27, 95% CI 0.18–0.41). 

#### 3.4.3. Living Situation

Younger age (AOR 0.68 95% CI 0.68–0.84), non-White racial status (AOR 3.19 95% CI 1.02–9.97), and less post-traumatic growth (AOR 0.97 95% CI 0.95–0.99) were significantly associated with living with parents. 

## 4. Discussion

The results from this study demonstrate significant differences for AYAs diagnosed with cancer compared to peers in attainment of employment and independent living. Moreover, 41% and 45% of our sample reported quitting school or work completely as a result of their cancer diagnosis. There remains a significant period of time during which AYAs diagnosed with cancer are significantly different than their same-age peers with respect to education and independent living. The long-term consequences of unemployment and dependent living for these youth diagnosed with AYA cancer would be significant.

The implications of unemployment status are far-reaching. Employment is important for enhancing an individual’s sense of usefulness and belonging [16]. Indeed, employment status was related to both physical and mental quality of life among AYAs diagnosed with cancer. In addition, employment is necessary to provide financial means. Previous research conducted by our research team has demonstrated the significant financial implications associated with being an AYA diagnosed with cancer, including being more likely to have outstanding credit card and line-of-credit balances, having higher rates of payday loans, being less likely to own a home, and more likely not to own assets [17]. The significant differences in employment status between groups noted until 45 years of age will significantly impact their financial well-being, as well as their quality of life, for many years to come. This may be especially true for survivors of head and neck cancers known to have received harsh treatments (i.e., chemotherapy, radiation therapy) directed at the central nervous system, which contribute to multiple psychosocial and functioning deficits for their entire lives [18]. 

Living status is also likely linked to employment status whereby AYAs diagnosed with cancer who are not working are unable to afford living alone. In our sample, AYAs diagnosed with cancer were significantly more likely to be living with their parents than comparisons. Living at home might also reflect the need for AYAs diagnosed with cancer to rely on parents to provide medical care and support. In our sample, non-White AYAs diagnosed with cancer were related to living at home. Culture may play a role in the resilience and protective factors for AYAs [19,20], and based on this premise, it is possible that non-White AYAs stay as a cohesive unit within the family following a major stressor/life event rooted in values of collectivism. For these AYAs, it may be a protective factor/positive outcome to be living at home. Nevertheless, our data suggest that those who living at home report lower levels of post-traumatic growth. Alternatively, this finding may indicate that the financial implications of a cancer diagnosis for young people have greater consequences for those who are non-White. Certainly, we know that the concepts of race and ethnicity, and socioeconomic status, are intertwined and inseparable [11,21], and that those who are impoverished experience additional impairments after cancer treatment [22,23]. As a society, we have an obligation to ensure that all AYAs, regardless of socioeconomic status, race, or ethnicity, have an equal chance to succeed after their cancer diagnosis. Greater diversity within our sample is needed to more fully explore these questions. 

AYAs diagnosed with cancer were more likely to be in a relationship than comparisons, although this difference seemed to exist only for youth 20–29 years of age. Nevertheless, this difference was contrary to the previously published literature. For example, in a study examining a large population sample of AYAs diagnosed with cancer from the US, AYAs were less likely to be married and at an increased risk of divorce/separation than controls [8]. Our findings were different from the published literature but may be explained by previous research that has described changes in life perception and values, particularly around marriage, that can accompany a cancer diagnosis for young people [24]. In our study, those who were single reported worse mental quality of life, post-traumatic growth and social support, and therefore relationship status may confer a protective factor for these AYAs. These findings lend further evidence for the importance of peer support programs and the need for youth to feel connected to others. 

The implications of this work are multiple. Clinically, these results should support advocacy efforts for more specialized follow-up care for AYAs diagnosed with cancer. Although guidelines exist which promote the need for regular surveillance of education and employment outcomes among childhood, adolescent, and young adult survivors of childhood cancer [5], it is unclear for these young adults who within their healthcare teams would be responsible for this routine monitoring. In addition, while “return to work programs” exist, few have been designed and tailored specifically for AYAs diagnosed with cancer [25]. Future research should explore the adaptation of such programs for these young people. 

Although we believe there are many strengths to this study, we appreciate that there are some limitations as well. We recognize that the sample included may not be representative of all Canadian AYAs. First, the sex distribution within our sample was significantly skewed in favor of females (83%). Although we did try to account for this skewed distribution in our approach of matching the comparison sample to the YACPRIME sample by sex and age at a ratio of 1:3, it is important to note how the larger proportion of females may have impacted our data. We also acknowledge that the distribution of diagnosis in our sample does not necessarily reflect the distribution of diagnoses seen in the broader cancer population. Specifically, our sample included a lower percent of melanoma patients and a higher rate of patients diagnosed with gastrointestinal cancers. Another limitation of this work is the method by which our sample was recruited; that is, through the patient advocacy organization Young Adult Cancer Canada (YACC). Although participants were asked whether they had ever been involved with YACC, only 36% reported that they had not; it is possible that these youth are qualitatively different from those not engaged with the same organization. Given the cross-sectional nature of our work, it is impossible to determine causality among relationships. Finally, it should be noted that the questions for the social status items were not equivalent (i.e., word for word) between the two surveys. Given these limitations, the results of the study should be interpreted with caution. Future research may consider age at diagnosis and era of treatment as factors that could play a role in social status. 

## 5. Conclusions

Adolescence and young adulthood represent a time during development when major transitions can take place. For AYAs diagnosed with cancer, this can be a particularly difficult and vulnerable time as they undergo diagnosis and treatment while navigating developmental changes. The interaction of these processes can implicate one’s sense of independence, relationships, and school and career development. This study sheds light on some of the ways in which the demographic profile and psychosocial functioning of AYAs diagnosed with cancer can play a role in their social status, including their employment and relationship status, over the course of their developmental trajectory. 

## Figures and Tables

**Table 1:** Descriptive characteristics of the sample.

	AYA (*n* = 622)	Comparison (*n* = 1688)	*p*
	*n* (%)	*n* (%)	
Characteristic			
Sex			1.00
Male	84 (13.5)	221 (13.1)	
Female	537 (86.3)	1467 (86.9)	
Age at Time of Study			1.00
20–29 years	131 (21.1)	363 (21.5)	
30–39 years	377 (60.6)	1019 (60.3)	
40–49 years	105 (16.9)	282 (16.7)	
50–64 years	9 (1.4)	24 (1.4)	
Income (CAD)			
<CAD 40,000	135 (21.7)	389 (23.0)	
CAD 40,000–80,000	164 (26.4)	485 (28.7)	
>CAD 80,000	273 (43.9)	814 (48.2)	
Missing	50 (8)	0 (0)	
Province of Residence			
Alberta	101 (16.2)	279 (16.5)	
British Columbia	90 (14.5)	249 (14.8)	
Manitoba	37 (5.9)	98 (5.8)	
New Brunswick	11 (1.8)	33 (2.0)	
Newfoundland and Labrador	66 (10.6)	163 (9.7)	
Northwest Territories	1 (0.2)	3 (0.2)	
Nova Scotia	34 (5.5)	101 (6.0)	
Nunavut	0 (0)	0 (0)	
Ontario	195 (31.4)	561 (33.2)	
Prince Edward Island	5 (0.8)	12 (0.7)	
Quebec	65 (10.5)	139 (8.2)	
Saskatchewan	15 (2.4)	(45 (2.7)	
Yukon	2 (0.3)	5 (0.3)	
Race/Ethnicity			
White	543 (87.3)	1489 (88.2)	
Non-White	79 (12.7)	150 (8.9)	
Asian	21 (3.4)		
Multi-racial	25 (4.0)		
Aboriginal/First Nations	13 (2.1)		
Other	20 (3.2)		
Diagnosis			
Breast	170 (27.3)		
Female Genitourinary	60 (9.6)		
Male Genitourinary	9 (1.4)		
Thyroid	45 (7.2)		
Blood	173 (27.8)		
Head and Neck	46 (7.4)		
Gastrointestinal	59 (9.5)		
Skin	18 (2.9)		
Other	34 (5.5)		
Multiple Types	8 (1.3)		
Diagnosis Stage			
Stage 1	85 (13.7)		
Stage 2	170 (27.3)		
Stage 3	145 (23.3)		
Stage 4	87 (14.0)		
Don’t Know/Not Applicable	135 (21.7)		
Treatment			
Surgery	261 (42.0)		
Chemotherapy	267 (42.9)		
Radiation	183 (29.4)		
Age at Diagnosis, years (mean, SD)	29.05 (5.99)		
% on Treatment	184 (29.6)		
Time Since Treatment, years (mean, SD)	4.45 (5.42)		
Education (*n* = 613)			0.93
Part-time Student	7 (1.1)	43 (2.5)	
Full-time Student	37 (5.9)	87 (5.2)	
Other	536 (86.2)	1557 (92.2)	
Employment (*n* = 613)			<0.001
Working Part-time	95 (15.3)	190 (11.3)	
Working Full-time	256 (41.2)	1113 (65.9)	
Other	271 (43.6)	385 (22.8)	
Relationship (*n* = 613)			<0.09
Single	171 (27.5)	526 (31.2)	
Married/Common-Law	415 (66.7)	1020 (60.4)	
Widowed/Separated/Divorced	30 (4.8)	138 (8.2)	
Other		4 (0.2)	
Living Situation (*n* = 613)			<0.001
Living with Parents	74 (11.9)	112 (6.6)	
Living Alone	99 (15.9)	314 (18.6)	
Living Alone with Children	31 (5)	175 (10.4)	
Living with Others	175 (28.1)	291 (17.2)	
Living with Others and Children	194 (31.2)	696 (41.2)	
Other	49 (8.0)	95 (5.6)	

**Table 2:** Multivariable logistic regression analyses of characteristics associated with social status.

	Employment Status		Relationship Status		Living Situation	
Variable	Adjusted Odds Ratio (AOR)	95% CI	Adjusted Odds Ratio (AOR)	95% CI	Adjusted Odds Ratio (AOR)	95% CI
Sex						
Male	reference		reference		reference	
Female	1.37	0.67–2.81	0.94	0.40–2.20	1.27	0.36–4.51
Current Age	1.07	1.02–1.12	0.89	0.84–0.94	0.75	0.68–0.84
Race/Ethnicity						
White	reference		reference		reference	
Other	1.41	0.66–3.02	0.99	0.41–2.36	3.19	1.02–9.97
Cancer Diagnosis						
Blood	1.54	0.81–2.93	1.59	0.78–3.22	1.55	0.54–4.45
Other	reference		reference		reference	
Chemotherapy						
Yes	0.68	0.37–1.27	1.78	0.83–3.81	2.49	0.65–9.95
No	reference		reference		reference	
Metastatic Status						
Yes	reference		reference		reference	
No	3.23	1.08–9.62	1.03	0.32–3.35	-	-
Physical Component	1.07	1.04–1.10	0.98	0.95–1.01	1.01	0.96–1.06
Mental Component	1.06	1.03–1.09	1.04	1.01–1.07	1.03	0.99–1.08
Post-traumatic Growth	1.00	0.98–1.01	1.01	1.00–1.03	0.97	0.95–0.99
Social Support	0.89	0.65–1.21	0.27	0.18–0.41	1.23	0.70–2.28

## Data Availability

The data that support the findings of this study are available from the corresponding author upon reasonable request.
